# Immunologic activation of human syncytiotrophoblast by *Plasmodium falciparum*

**DOI:** 10.1186/1475-2875-7-42

**Published:** 2008-02-29

**Authors:** Naomi W Lucchi, David S Peterson, Julie M Moore

**Affiliations:** 1 Department of Infectious Diseases and Center for Tropical and Emerging Global Diseases, University of Georgia, Athens, GA 30602, USA; 2 Centers for Disease Control and Prevention, 4770 Buford HYW, MS-F12 Chamblee, Georgia, 30341, USA

## Abstract

**Background:**

Malaria during pregnancy is characterized by the sequestration of malaria-infected red blood cells (iRBC) in the intervillous spaces of the placenta, often accompanied by the infiltration of maternal mononuclear cells, causing substantial maternal and foetal/infant morbidity. The iRBC bind to receptors expressed by the syncytiotrophoblast (ST). How ST responds to this interaction remains poorly understood. Because it is known that ST is immunoactive and can respond to infectious agents, the consequences of this ST-iRBC interaction should be investigated.

**Methods:**

An in vitro system was used to assess the biochemical and immunological changes induced in ST by ST-adherent iRBCs. Changes in ST mitogen-activated protein kinase (MAPK) activation were assessed by immunoblotting and mRNA expression levels of selected cytokine and chemokines in primary ST bound by iRBC were determined using real-time, reverse transcription PCR. In addition, secreted cytokine and chemokine proteins were assayed by standard ELISA, and chemotaxis of PBMC was assessed using a two-chamber assay system.

**Results:**

Following iRBC/ST interaction, ST C-Jun N-terminal kinase 1 (JNK1) was activated and modest increases in the mRNA expression of TGF-β and IL-8/CXCL8 were observed. In addition, this interaction increased secretion of MIF and MIP-1α/CCL3 by ST and induced migration of PBMC towards iRBC-stimulated ST.

**Conclusion:**

Results from this study provide the first evidence that ST participates in shaping the local immunological milieu and in the recruitment of maternal immune cells to the maternal blood space during placental malaria infection.

## Background

It is estimated that annually approximately 2.2 billion people are exposed to the risk for *Plasmodium falciparum *malaria infection and between 300–600 million clinical attacks are attributable to this parasite [1]. Ninety percent of deaths occur in sub-Saharan Africa, the majority involving children less than five years of age. In addition to children, pregnant women (particularly those in their first pregnancy) are at highest risk of severe disease [2]. A hallmark of malaria during pregnancy is the sequestration of malaria-infected red blood cells (iRBCs) containing late developmental stages in the intervillous spaces (IVS) of the placenta [3-5]. This is usually accompanied by the infiltration of maternal leukocytes, especially monocytes, in the IVS [6,7] and haemozoin deposition [4,8], resulting in what is referred to as placental malaria (PM). PM poses substantial risk to the mother, the foetus, and the neonate in the form of maternal anaemia and poor foetal outcomes such as low birth weight (LBW) and prematurity ([9,10]; reviewed in [11,12]).

The sequestration of iRBCs in the placenta is thought to be mediated in large part by the cytoadherence of iRBCs to placental receptors expressed in the IVS and on the syncytiotrophoblast (ST; foetal epithelial cells that are in direct contact with maternal blood within the IVS). Currently, it is believed that the glycosaminoglycan chondroitin sulfate A (CSA) is the principal placental iRBC receptor [13-15]. Other minor receptors are proposed to exist [16-19], although the role of hyaluronic acid [20] has recently been questioned [15]. Parasite-encoded surface ligands expressed on the membrane of iRBCs are thought to facilitate this adherence. To date, the only well-studied cytoadherence parasite protein is the *P. falciparum *erythrocyte membrane protein-1 (PfEMP1) encoded by the highly polymorphic members of the *var *gene family [21,22]. The most well characterized PfEMP1 variant identified to mediate iRBC binding to the placenta is VAR2CSA [23-25]. Despite intense effort to elucidate the placental host/parasite interaction on the molecular level, the consequences of this placental iRBC sequestration on ST cell function have largely been ignored.

The immunological consequences of malaria in pregnancy have been widely investigated. A protective IgG antibody response that blocks the binding of iRBC to CSA in the placenta has been shown to develop in a sex- and gravidity-dependent manner [26,27]. In addition, several studies have demonstrated the presence of both proinflammatory and anti-inflammatory immune factors in malaria-infected placentas [28-31]. For example, increased amounts of Th1 cytokines such as TNF-α [29,31], IFN-γ [29,30] and IL-1β [31] have been demonstrated in PM-positive placental blood. Production of IL-10 by intervillous blood mononuclear cells (IVBMC) was also shown to be increased in PM [28,30] and was hypothesized to be important in the control of the negative effects of Th1 cytokines on pregnancy [28-31]. In addition, several proinflammatory chemokines have been observed in association with PM including interleukin-8 (CXCL8/IL-8) [31,32] and beta chemokines such as macrophage inflammatory protein-1 alpha (MIP-1α/CCL3), macrophage chemoattractant protein-1 (MCP-1/CCL2), I-309/CCL1 [32] and MIP-1β/CCL4. [33]. Massively elevated levels of macrophage migration inhibitory factor (MIF) were observed in the placental plasma from women with PM [34], and immunohistochemical analysis suggests that ST contributes to this increase, particularly in cases of PM (36). However, in most cases, the cell types responsible for the production of these immune factors have not been definitively identified, although cells of both foetal and maternal origin have been implicated[31,32,35]. While proinflammatory responses are important in the clearance of iRBCs and protection against PM, they have also been shown to play a major role in malaria pathophysiology and contribute to morbidity [36]. These proinflammatory immune responses have been associated with the ensuing monocytic infiltration [6,32] which has been associated with poor foetal outcomes [6,7,9]

Foetally-derived tissues have been demonstrated to be active participants in maintaining the immunological milieu at the maternal-foetal interface during pregnancy [37]. However, their role during PM remains largely unknown. Furthermore, the consequences of iRBC sequestration in the IVS on the function of the immunologically active ST are not known. This has partly been due to the lack of a good in vitro model system with which to investigate this. Previous studies utilizing such a system [18] showed that the ST is capable of responding to iRBC binding via pan-tyrosine phosphorylation of ST proteins [18]. In the present study, the impact of iRBC interaction/binding on ST cells was further investigated. Activation of immunologic signaling in ST was observed upon stimulation with *P. falciparum *infected-RBC selected for binding to ST. This activation was found to be associated with changes in cytokine and chemokine expression and secretion. Furthermore, this iRBC/ST interaction stimulated the chemotactic migration of peripheral blood mononuclear cells (PBMC).

## Materials and methods

### Trophoblast and parasite culture

Trophoblast isolation and cell cultures were performed as previously described [18]. Briefly, BeWo cells were grown in minimum essential medium and induced to form ST (BeWo^ST^) by use of 40 uM forskolin. Primary placental cytotrophoblast cells (CT) were isolated from fresh human placentas obtained from women delivering at Athens Regional Medical Center, Athens, Georgia, USA. Placentas were obtained with written, informed consent under approval by the University of Georgia and Athens Regional Medical Center Institutional Review Boards. The immunopurified cells were used immediately or cryopreserved in liquid nitrogen until use.

*Plasmodium falciparum *was cultured and selected for binding to ST as described elsewhere [18]. Briefly, the FCR3 (Malaria Research and Reference Reagent Resource Center (MR4)) laboratory strain was sequentially panned on ST to select for ST-binders (iRBC^ST^). The CS2 isolate (MR4), known to bind to CSA, was also included. The parasites were synchronized by freezing and thawing or by sorbitol purification [38] and experiments performed using the FCR3 selected cultures consisting largely of mature trophozoite and schizont stages that bind to ST. The parasite cultures were routinely tested and found negative for mycoplasma contamination by PCR. All experiments used FCR3 except where noted.

### Immunoblotting

BeWo^ST ^were incubated with iRBC^ST ^for given time points after which whole-cell lysate extracts were obtained as described [18]. Unstimulated cells or cells incubated with uninfected RBC (uRBCs) were included in the assays as controls. Immunoblotting was carried out as described earlier [18] with a few changes. The membranes were probed with primary antibodies against phosphorylated extracellular signal-regulated kinase 1/2 (ERK1/2), C-Jun N-terminal kinase 1 (JNK1) and p38 (Cell Signaling Technology Inc., Beverly, MA) as recommended by the manufacturer. Final detection was performed with appropriate horseradish peroxidase-labelled (HRP) secondary antibodies (Sigma St. Louis, MO) in blocking buffer for one hour at room temperature. Phosphorylated proteins were visualized by enhanced chemiluminescence (SuperSignal, Pierce, Rockford, IL). Each membrane was stripped with freshly prepared stripping buffer (2% SDS; 62.5 mM Tris-HCl, pH 6.7; 100 mM 2-mercaptoethanol) and reprobed in a similar fashion with antibodies to the housekeeping gene, β-actin (Sigma, St. Louis, MO). The latter was used as a loading control and for densitometric analysis performed using QuantityOne software (Bio-Rad, Hercules, CA).

### Real-time PCR

Total RNA was isolated from primary ST using the RNeasy Qiagen kit (Qiagen, Valencia, CA) following the manufacturer's protocol and stored at -85°C. Contaminating genomic DNA (gDNA) was digested using RNAse-free DNase (Ambion Inc. Austin, TX) as recommended by the manufacturer. First strand cDNA was synthesized from 1 μg of total RNA using the Omniscript reverse transcription kit (Qiagen, Valencia, CA). Real-time PCR was carried out using specific primers for TNF-α, TGF-β, IL-8/CXCL8, IL-10, MIF, MCP-1/CCL2, IFN-γ and 18S ribosomal RNA (Table 1) (all from MWG-Biotech Inc., High Point, NC). All primers were validated for use in comparative real-time PCR. Real-time PCR was performed using the Mx3000P thermocycler and programme (Stratagene, La Jolla, CA). No template controls and no reverse transcription controls were included. The 2^ΔΔ CT method of analysis was used with the 18S RNA gene as normalizing gene. Each sample was analysed in duplicate. Results from three different experiments are given as mean fold increase over uRBC-stimulated ST cells.

**Table 1:** Oligonucleotide primers used in the amplification of the genes

**Primer Name**	**Sequence**
IL-8 Forward	5'-GCCAAGGAGTGCTAAAGAAC-3'
Il-8 Reverse	5'-TCCATCAGAAAGCTTTACA-3'
TNF-α Forward	5'-GAGCACTGAAAGCATGATCCG-3'
TNF-α Reverse	5'-AGCAGGCAGAAGAGCGTGGT-3'
MCP-1 Forward	5'-CAATCAATGCCCCAGTCACC-3'
MCP-1 Reverse	5'-GGAGTTTGGGTTTGCTTGTC-3
TGF-β Forward	5'-TACCAGAAATACAGCAACAAT-3'
TGF-β Reverse	5'-CTCCACGGCTCAACCACTG-3'
IFN-γ Forward	5'-GCATCGTTTTGGGTTCTCTTG-3'
IFN-γ Reverse	5'-TCCATTATCCGCTACATCTGAA-3'
IL-10 Forward	5'-GCACCCACTTCCCAGGCAA-3'
IL-10 Reverse	5'-GAAGGAATCATACTCACAAAGAAAG-3'
MIF Forward	5' CCACCGGCAAGCCCCCCCA-3'
MIF Reverse	5'-TGTAGGAGCGGTTCTGCG-3'
18 S Forward	5'-GTAACCCGTTGAACCCCATT-3'
18S Reverse	5'-CCATCCAATCGGTAGTAGCG-3'

### Cytokine and chemokine ELISA

Primary ST were stimulated over a 24 hour time course with iRBC^ST^, uRBCs or left unstimulated. Supernatants were collected from the stimulated cells and stored until use. As a control, wells containing uRBCs or iRBC^ST ^with no ST were included in the experiments and supernatants collected. A standard cytokine sandwich ELISA was performed according to the manufacturer's protocol (R&D Systems, Inc. Minneapolis, MN) for TNF-α, TGF-β, IL-10, MIF, MIP-1α/CCL3, MIP-1β/CCL4, and IL-8/CXCL8. ELISA results for TGF-β were inconsistent and difficult to interpret and thus were not included in the analysis.

### Chemotaxis assay

Peripheral blood was obtained from healthy female American volunteers by a trained phlebotomist. An equal part of sterile phosphate buffered saline (PBS) was added to the blood and this was layered on fico-lite LymphoH (Atlanta Biological, Atlanta, GA) in a 50 ml centrifuge tube. To obtain PBMCs, the tube was centrifuged at 355 × g for 30 minutes at 25°C. The interface, containing the PBMCs, was collected and washed twice with sterile PBS. The cells were counted and used for the chemotaxis experiments. ST cells were grown in 24-well plates (BD Biosciences, Franklin Lakes, NJ) and stimulated with iRBC^ST ^or uRBCs for 12 hours or left unstimulated. PBMCs were used at 5 × 10^5 ^cells/ml. 3-μm pore size cell culture inserts (BD Biosciences, Franklin Lakes, NJ) were placed into each well and 350 μl of PBMC were added and allowed to migrate for 12 hours. The inserts were removed and placed in new wells. Using a cotton swab the inserts were carefully wiped to remove cells that had not migrated. Migrated cells, on the underside of the membranes, were stained with calcein-AM (Molecular Probes, Eugene, OR) for 30 minutes at 37°C and washed 3 times. The number of migrated cells was then determined by counting eight random fields under an inverted fluorescence microscope at 20× magnification.

### Statistical analysis

A non-parametric Friedman's test was used to determine differences in the chemokine production and differences in the CT values for the real-time PCR. For chemotaxis experiments, the differences in number of migrated cells upon stimulation with the different stimuli were analysed using a student's t-test.

## Results

### Stimulation of JNK1 in ST cells by binding of iRBC^ST^

**Figure 1:**
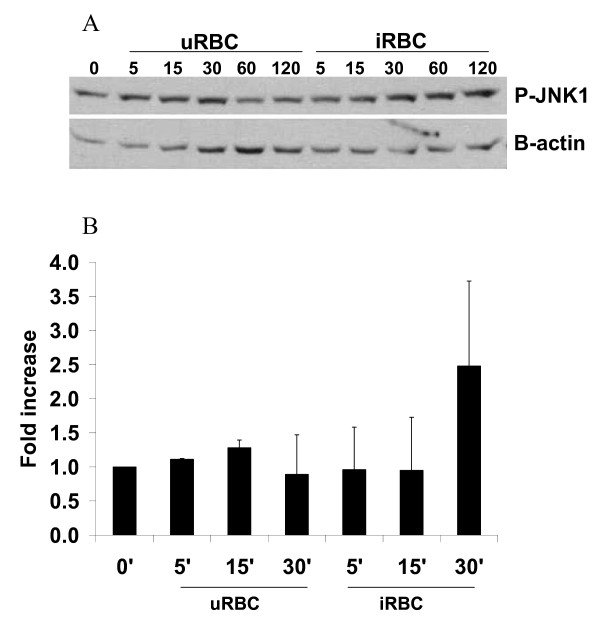
**Activation of JNK following iRBC^ST ^binding**. BeWo^ST ^cells were co-cultured with iRBC^ST ^or uRBC for the indicated time course or left unstimulated (0). Panel A represents a western blot analysis of cellular proteins from ST cells following stimulation. Densitometry analysis of the western blot results from two experiments was performed. Figure B shows specific enhanced phosphorylation (~2.5 fold) of JNK1 (p-JNK1) after 30 minutes of co-culture with iRBC^ST^. Co-culture with uRBCs led to a slight increased phosphorylation (< 1.5 fold increase over unstimulated) after 15 minutes. Data shown represent mean ± SD fold increase relative to unstimulated cells from two separate experiments.

Increased phosphorylation of JNK1 (~2.5 fold increase) was evident after 30 minutes incubation of BeWo^ST ^with iRBC^ST ^(Figure 1). Incubation with both uRBC and iRBC^ST ^demonstrated slight increases in JNK1 activation (< 1.5 fold increases) within 5–15 minutes, but a greater than 2 fold increase was only observed in the presence of iRBC^ST ^30 minutes post stimulation. These stimulation conditions did not lead to changes in ERK1 nor p38 mitogen-activated protein kinases (MAPKs).

### Gene expression changes in ST stimulated with cytoadherence iRBC^ST^

**Figure 2:**
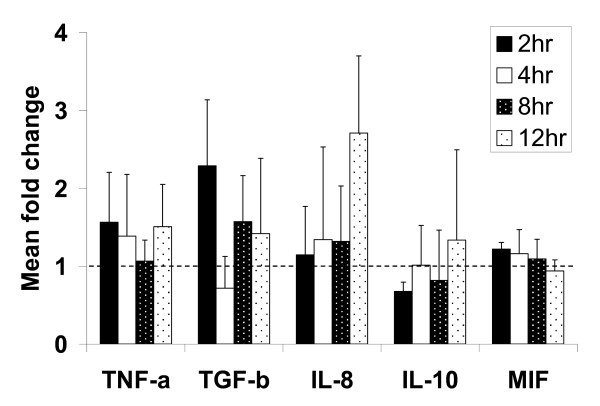
**Gene expression changes in ST stimulated with iRBC^ST^**. Primary ST was stimulated with either uRBC or iRBC^ST^. RNA was extracted and gene expression changes assayed by real time PCR. This treatment led to the marginal, but not statistically significant, upregulation of both TGF-β and IL-8/CXCL8 which showed more than two-fold increases in their mRNA expression at two hours and 12 hours, respectively. TNF, IL-10 and MIF mRNA did not change considerably with this treatment. Dotted line shows gene expression of uRBC-stimulated cells. Mean ± SD from three placentas is shown.

To determine if the MAPK activation resulted in gene expression changes, several factors were selected for analysis by real-time PCR (Figure 2). Following in vitro stimulation of primary ST with iRBC^ST ^compared to stimulation with uRBC, greater than two-fold increases were observed in the mRNA expression of TGF-β (at two hours) and IL-8/CXCL8 (12 hours post stimulation) but not at the other time points. However, none of the observed differences reached statistical significance (p > 0.05). Major changes in the mRNA levels of TNF-α, MIF and IL-10 were not evident (Figure 2). Expression of the genes encoding IFN-γ and MCP-I/CCL2 was not detected under any conditions.

### Cytokine secretion upon stimulation with iRBC^ST^

**Figure 3:**
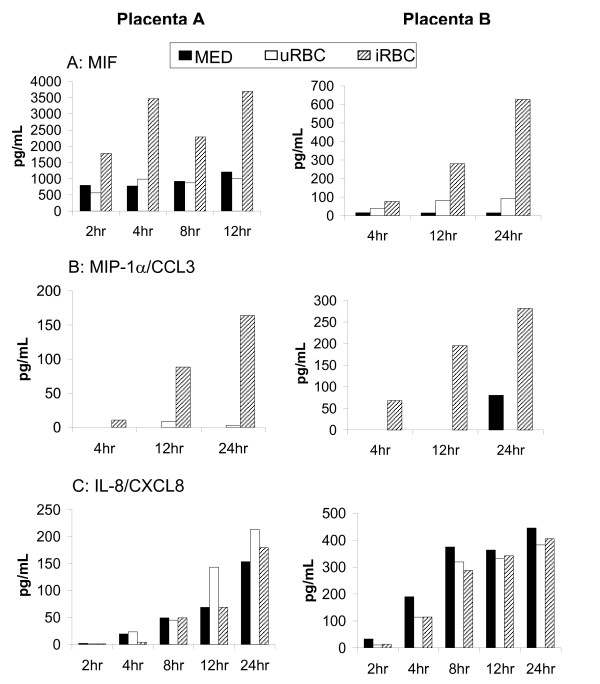
**Chemokine Secretion by primary ST cells upon the binding of iRBC^ST^**. Primary ST was stimulated with iRBC^ST ^(iRBC), uRBCs or left unstimulated (MED) over given time courses as indicated. (A) While stimulation with uRBC did not lead to any increase in MIF secretion more than that observed for unstimulated ST, stimulation with CS2-iRBC^ST ^led to an increased time-dependent secretion. This difference in MIF production among the different stimulant was statistically significant (p < 0.0032). (B) Two out of five placentas tested showed a time dependent increase in MIP-1α/CCL3 secretion upon interaction with iRBC^ST ^but not with uRBC or unstimulated this difference being statistically significant (p < 0.0040). (C) Secretion of IL-8/CXCL8 increased over time even in unstimulated cells, with no significant additional increase with iRBC^ST ^stimulation. Independent results from two placentas are shown.

In addition to gene expression analysis, functional ST activation was assessed via the measurement of cytokine and chemokine secretion. Supernatants from stimulated primary ST were used. Similar to BeWo^ST ^[35], substantial time-dependent secretion of MIF was observed upon stimulation of primary ST with iRBC^ST ^but not with uRBC or unstimulated cells (Figure 3A). The differences in MIF secretion were significantly different among the stimuli (p < 0.0032). MIP-1α/CCL3 was not consistently detected, but in 2 of 5 ST cultures, secretion was upregulated in response to iRBC^ST ^(Figure 3B) (p < 0.0040). The production of IL-8/CXCL8 did not increase with iRBC^ST ^binding, although a time-dependent increase, clearly representing constitutive expression, was observed (Figure 3C). No secretion of TNF-α, MIP-1β/CCL4, or IL-10 was detected.

### PBMC migrate toward iRBC^ST ^-stimulated ST cells

**Figure 4:**
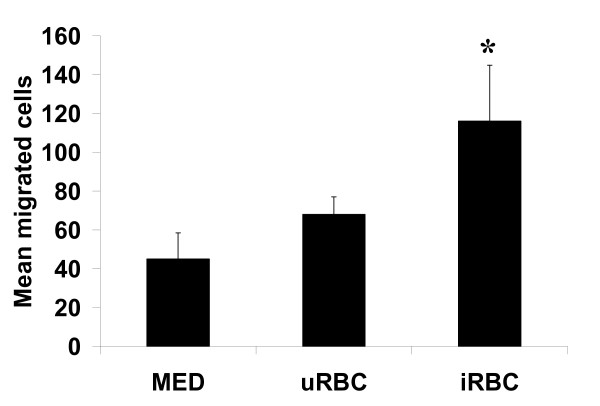
**Migration of PBMC towards iRBC^ST ^-stimulated ST cells**. Primary ST was stimulated with iRBC^ST^, uRBC or left unstimulated for 12 hours. PBMCs added to the upper chamber of a dual chamber were allowed to migrate for 12 hours. Calcein AM-stained cells in 8 random fields for each stimulant were counted. The mean ± SD of migrated cells upon stimulation was compared to the spontaneous migration towards unstimulated ST (MED). Interaction with iRBC^ST ^led to a significant migration of cells towards iRBC^ST^-stimulated ST compare to both controls (* = P < 0.0040). Representative data from one of two experiments is shown.

Given that iRBC^ST^-stimulated ST is capable of secreting chemokines, potentially including others not assayed in this study, it was critical to ascertain whether this secretion was sufficient to induce the migration of leukocytes. Using a two-chamber system, increased migration of PBMCs toward iRBC^ST^-stimulated ST cells (mean number of cells ± SD: 116 ± 29) compared to uRBC-stimulated (68 ± 9) or unstimulated ST (45 ± 13) was observed (Figure 4). The difference between the mean number of migrated cells upon uRBC and iRBC^ST ^interaction was statistically significant (p < 0.004).

## Discussion

Results from this study demonstrate for the first time that the ST is capable of responding immunologically to interaction with *P. falciparum*-infected RBCs selected for binding to ST. This was evidenced by the induction of MAPK pathways, secretion of immune factors such as MIF and MIP-1α/CCL3, and induction of migration of PBMC towards iRBC^ST^-stimulated ST.

The activation of MAPK pathways via phosphorylation is initiated by a large variety of external signals and leads to a wide range of cellular responses, including growth, differentiation, inflammation, apoptosis (reviewed in [39]), and secretion of cytokines (reviewed in [40]). This study demonstrates that the interaction of iRBC^ST ^with ST cells leads to the activation of the JNK1 pathway. In a previous study, the binding of iRBC^ST ^to ST cells was shown to result in the tyrosine phosphorylation of at least two ST proteins [18], altogether suggesting that a number of components of cellular activation pathways are activated in ST in the context of PM.

Indeed, the ST is known to be immunoactive, secreting cytokines and chemokines important for the maintenance of pregnancy [37] and in response to bacterial infections [41,42] and lipopolysaccharide [43]. The interaction with iRBC^ST ^led to the secretion of MIF and MIP-1α/CCL3 by ST. MIP-1α/CCL3 levels were shown to be increased in placental plasma of PM-positive women [32], although not so in another study [33]. The current study suggests that the ST may contribute to elevated MIP-1α/CCL3 levels in the placenta during PM. However, dramatic variation in the magnitude of the secretion of MIP-1α/CCL3 (~300 pg/ml of MIP-1α/CCL3 from one placenta preparation to below detectable levels in others) was observed. This is not unprecedented as other studies using isolated trophoblast cells from term placentas have reported variability in receptor expression on the trophoblast cells [44] and cytokine/chemokine secretion [45] among the different placenta preparations utilized.

Previous studies demonstrated that PM resulted in increased levels of MIF in the intervillous blood [34] and that BeWo cells, a trophoblastic cell line, secreted substantial amounts of MIF upon iRBC^ST ^interaction [35]. Together, these studies implicate the ST as an important source of MIF. This cytokine is known to play an important biologic role during pregnancy [46]. Importantly, it is also known to be involved in macrophage activation [47] and retention, as it inhibits macrophage migration. Therefore, MIF may play a role in the retention of maternal immune cells that may be recruited to the IVS by other chemokines such as MIP-1α/CCL3 and RANTES/CCL5 (unpublished data), leading to the increased levels of monocytes in the IVS during PM. Previous studies have shown that PM is commonly associated with monocyte recruitment to the IVS [6,7] which, in turn, is associated with high levels of intervillous chemokines [32]. Results from this study suggest that the ST contributes to the migration of immune cells into the IVS. It remains to be determined, however, what cell types are specifically migrating toward iRBC-stimulated ST and whether this activity contributes to protective or pathogenic effects in vivo.

Both IL-8/CXCL8 [31,32] and TNF-α have been associated with PM in previous studies. In this study, however, ST constitutively secreted IL-8/CXCL8 in a stimulation-independent manner and did not secrete any detectable TNF-α. This implies that the increased levels of IL-8/CXCL8 and TNF-α observed in PM-positive placentas are not derived from ST.

Variability in the secretion and gene expression of the investigated chemokines and cytokines between ST preparations from individual donors was observed. This reflects the inherent variability of human-based immunologic studies. In this context, it is important to keep in mind that differences in host genetic backgrounds contribute to the heterogeneities of malaria morbidity and disease manifestations [48-52]. Indeed, some cases of PM are accompanied by a massive infiltration of maternal mononuclear cells into the IVS but not others. It is currently unknown if these different disease manifestations are the result of variations in chemokine levels in the IVS which are driven by host genetic polymorphisms.

The experimental design utilized in the current study does not allow one to categorically claim that the effects observed were all directly due to the binding of iRBC^ST^. Both late stage trophozoites and schizonts (early and mature) were used to stimulate the ST and this co-culture was incubated for up to 24 hours in some experiments. Especially at late time points, some of the schizonts would have ruptured, releasing both haemozoin and glycosylphosphatidylinositol (GPI) among other host- and parasite-derived materials. Thus, it is possible that these malarial products also stimulated the ST cells. However, this system is not physiologically irrelevant. On the contrary, this complex mixture of malarial products and iRBCs is precisely what the ST cells experience in vivo, and, in fact, ongoing unpublished work suggests that haemozoin is a potent stimulator of ST immunologic responses (N. Lucchi et al., unpublished data). Nonetheless, further experiments including controls such as unselected iRBC or *var2csa*-null *P. falciparum *would reveal the extent to which the hypothesized points of iRBC^ST^/ST interaction (i.e., *var2csa*-encoded PfEMP1 and CSA) are required for ST activation. Furthermore, determining whether or not iRBC^ST ^binding affects non-immunologic ST functions such as nutrient and gaseous exchange could shed some light on the mechanisms leading to LBW associated with PM.

## Conclusion

This study provides the first evidence that the interaction of malarial parasites with ST cells in the placenta induces immunologic changes in the ST cells as evidenced by the activation of the MAPK pathways, upregulated secretion of MIF and MIP-1α/CCL3, and stimulation of PBMC chemotaxis. Thus, ST cells play an active immunological role in response to malarial parasites in the placenta and are capable of influencing and/or contributing to the local maternal immune environment.

## Authors' contributions

JMM, NWL and DSP conceived of the study and the study design and participated in data interpretation. The study was conducted by NWL. JMM and NWL drafted and critically reviewed the paper. DSP provided the parasites and maintained the parasite cultures together with NWL. All the authors reviewed and approved of the final version of the paper.
